# Determination of allograft fibrosis by measurement of liver stiffness using transient elastography in children after liver transplantation at Shiraz Organ Transplant Center 

**Published:** 2021

**Authors:** Seyed Mohsen Dehghani, Maryam Ataollahi, Seyyed Bozorgmehr Hedayati, Fateme Parooie, Iraj Shahramian

**Affiliations:** 1 *Shiraz Transplant Research Center, Shiraz University of Medical Sciences, Shiraz, Iran*; 2 *Gastroenterology Research Center, Shiraz University of Medical Sciences, Shiraz ,Iran*; 3 *Department of Pediatrics, Shiraz University of Medical Sciences, Shiraz, Iran*; 4 *Hematology research Center, Shiraz University of Medical Sciences, Shiraz, Iran*; 5 *Pediatric Gastroenterology and Hepatology Research Center, Zabol University of Medical Sciences, Zabol, Iran*

**Keywords:** Transient elastography, Liver transplantation, Allograft fibrosis.

## Abstract

**Aim:**

The aim of this study was to determine allograft fibrosis by measuring LS using TE in children after liver transplantation at Shiraz Organ Transplant Center.

**Background:**

Liver stiffness (LS) assessment using fibro-scanning (transient elastography-TE) is a non-invasive method for evaluating liver fibrosis.

**Methods:**

All children undergoing liver transplant from 2012 to 2016 were included in the study. Data on demographics, graft types, immunosuppressive drugs, as well as clinical and paraclinical data were obtained from patients’ records. TE was performed to determine LS in all patients. Liver fibrosis was also confirmed based on Metavir score.

**Results:**

During this period, more than 400 liver Tx were done in children, but only 54 patients, comprising 20 (37%) girls and 34 (63%) boys who underwent liver transplantation, were available and willing to participate in this study. The mean age of the patients was 12.96 ± 5.32 years. Correlations between FS score (LS) and AST (*p* = 0.01), total bilirubin (*p* = 0.002), albumin (*p* = 0.001), PT (*p* = 0.03), and INR (*p* = 0.001) were significant. There was no significant relationship between FS score (LS) and type of allograft (*p* = 0.79) and underlying disease (*p* = 0.36). Positive and significant correlations were observed between Metavir score and AST (*p* = 0.01), total bilirubin (*p* = 0.01), INR (*p* = 0.004), and cholesterol (*p* = 0.001). The severity of fibrosis significantly and negatively correlated with albumin (*p* = 0.004) and glucose (*p* = 0.003). Also, there was no significant relationship between Metavir score and allograft type (*p* = 0.7).

**Conclusion:**

The current study demonstrated that 14.9% of LT patients had a METAVIR ≥ F2. The time between LT and TE was significantly correlated with LS and the degree of liver fibrosis based on Metavir score. However, there was no significant relationship between LS with allograft type or underlying liver disease.

## Introduction

 Liver transplant (LT) is very successful in children, and most liver diseases are potentially treatable in children with LT. Nevertheless, quality of life and allograft function gradually decline in LT patients (1, 2). Several pediatric and adult centers have examined serial liver biopsies after liver transplant to evaluate long-term histological changes (3-6). Histological abnormalities are frequently observed in biopsies at 12 months after LT in pediatric allograft recipients. Many of them have been seen accompanied by apparently normal or near normal liver biochemistry and clinical parameters (7). The most common findings in liver allograft biopsies in children are idiopathic post-transplant hepatitis and graft fibrosis with severity of fibrosis increasing over time after LT (7-9). However, children with post-transplant idiopathic hepatitis or fibrosis are often asymptomatic with normal or near-normal liver biochemical tests (10).

Although the serial evaluation of bilirubin, alanine aminotransferase (ALT), aspartate aminotransferase (AST), and gamma glutamyl transferase (GGT) is recommended, the best time interval for testing is not defined and varies from one to three months. Moreover, these tests may not be able to reveal mild liver allograft impairment. 

Transient elastography (TE), or fibroscan, is a non-invasive procedure for estimating liver fibrosis and determining liver stiffness (LS) in patients with chronic liver disease. This procedure can be easily and quickly done bedside or in an outpatient clinic. This procedure is well tolerated by patients and widely available. In adult LT patients, this technique has been excellent for the diagnosis of liver fibrosis/cirrhosis; however, its efficiency may be lower in patients with mild fibrosis (11, 12). TE has also been an accurate predictor of liver fibrosis in children (13). Two pediatric studies have compared TE to histology after LT and confirmed the potential of this technique (14, 15). Nonetheless, this procedure is difficult to perform in split or partial grafts. The aim of this study was to evaluate allograft fibrosis in children receiving LT by TE in the Shiraz Organ Transplant Center. 

## Methods

**Patients**

The study population comprised all children younger than 18 years of age at the time of transplant who underwent LT at Shiraz Organ Transplant Center, affiliated with Shiraz University of Medical Sciences, from 2012 to 2016. Those who had died at one year after LT, were unwilling to participate, or did not routinely attend follow-up examinations were not included in the study.

**Exclusion criteria**

Patients who were very obese or had developed ascites which made elastography difficult as well as patients with cardiac devices were excluded. 

**Data collection**

Data on patient demographics, transplant indications, graft type, familial relationship with donor, immunosuppressive drugs, and clinical and paraclinical information including serum AST, ALT, GGT, total bilirubin, cholesterol, triglycerides, glucose, PT, INR, and albumin at the time of TE were extracted from the patients’ records. Written informed consent was obtained from all the patients’ parents. 

**Liver stiffness**

Liver LS was evaluated using TE, which is a method based on ultrasound. TE is equipped with a probe with an ultrasonic transducer mounted on a vibrating shaft. Light and low frequency vibrations are transmitted through the vibrator to the tissue through a stimulator. These vibrations create an elastic shear wave that propagates through the tissue. At the same time, ultrasonic pulse echo accumulation is followed by shear wave propagation and velocity measurements that are directly related to tissue hardness. The harder the tissue, the faster the shear wave propagates. Liver stiffness was measured by an experienced radiologist who was expert in performing and interpreting TE. The right position was evaluated to be on the anterior axillary line in the right intercostal space. The distance between the thoracic perimeter and the skin capsule determined which probe (out of three) should be used. The researcher explained to the children that TE was a painless procedure, and they were asked not to move during the procedure. LS was measured using Fibroscan1 with standard M and 3.5 MHz probes (7 mm in diameter). The patients were kept in a supine position with maximal right-hand abduction while undergoing abdominal ultrasound to detect areas of the liver lacking large vascular structures. The tip of the fibro-scan transducer was placed on the skin between the rib and the right lobe of the liver. 

The final LS results were obtained with a mean of 10 successful acquisitions. A successful procedure was considered when 6 out of 10 measurements were valid. Patients with unsuccessful examinations were excluded from the final analysis. 

CAP was recorded as the attenuated ultrasound wave of the liver at 3.5 MHz which was simultaneously measured along with LS and using the M probe. The final CAP value varied from 100 to 400 dB from an average of 10 valid measurements. For each patient, the success rate of the procedure was calculated as the ratio of the number of successful parameters to the total number of attempts (expressed as a percentage). 

**Statistical analysis**

The data was analyzed using SPSS 22 software. Descriptive statistics (frequency, percentage, mean ± standard deviation) were used for statistical description. The chi-square and Fisher exact tests were used to assess relationships between qualitative variables. Independent samples t-test, ANOVA, and Kruskal Wallis were used to compare quantitative findings between groups. Pearson correlation was applied to assess associations between quantitative variables. In this study, a *p*-value less than 0.05 was considered statistically significant. 

## Results

This study studied 54 patients, i.e. 20 (37%) girls and 34 (63%) boys, who had undergone LT. The mean age of the patients was 12.96 ± 5.32 years, and the youngest and oldest children were 4 and 24 years old, respectively. The mean weight of the patients was 39.99 ± 18.94 kg (ranging from 7 to 70 kg). The mean height was 130.62 ± 32.48 cm (ranging from 148 to 175 cm). TE was performed for all patients with 100% success rate. The mean FS score was obtained as 5.4 ± 74.23 kPa (ranging from 2.6 to 25.3 kPa). The mean CAP score was 148.54 ± 46.42 (ranging from 100 to 293).

According to the Metavir score, F0-F1 indicates mild fibrosis, F2 indicates moderate fibrosis, F3 indicates severe fibrosis, and F4 indicates cirrhosis. In the present study, 46 patients (85.2%) had mild fibrosis (F0-F1), 5 patients (9.3%) had moderate fibrosis (F2), and 3 patients (5.6%) had severe fibrosis. Table 1 shows the means of laboratory findings in the patients. 

The most common used immunosuppressive was tacrolimus (44/50), followed by mycophenolate (19/50), prednisolone (10/50), and sirolimus (7/44). The mean time between LT and TE was 6.31 ± 2.98 years (ranging from 2 to 18 years). Allograft types were whole organ in 38 (70.4%), partial in 12 (22.2%), and split in 4 (7.4%) patients.

**Figure 1:**
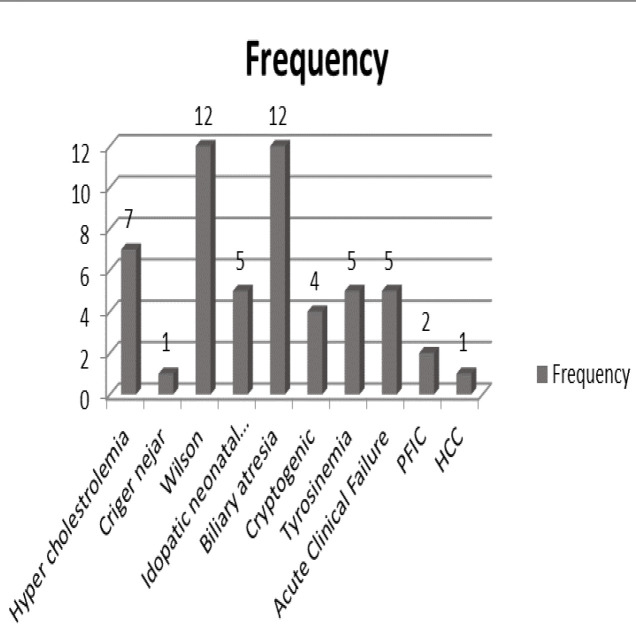
Underlying liver diseases in children who underwent liver transplant

Figure 1 shows the underlying liver diseases indicated for LT in this study. As can be seen, Wilson disease and biliary atresia were the most common underlying diseases.

Allografts were obtained from a first-degree relative (parents) in 12 (22.2%), a second-degree relative in 3 (5.6%), and a deceased donor in 39 (72.2%) patients. According to Pearson correlation analysis, there were no significant relationships between FS score and white blood cell count (*p* = 0.31), hemoglobin (*p* = 0.89), platelet count (*p* = 0.63), ALT (*p*=0.88), ALP (*p* = 0.56), direct bilirubin (*p* = 0.07), GGT (*p* = 0.79), TG (*p* = 0.31), and cholesterol (*p* = 0.18). However, the associations between FS score and AST (*p* = 0.01), total bilirubin (*p* = 0.002), albumin (*p* = 0.001), PT (*p* = 0.03), INR (*p* = 0.001), glucose (*p* = 0.002) and the time between LT and TE (*p* = 0.02) were significant.

**Table 1:** Laboratory variables in children with liver transplantation

Variables	Mean ±SD	Maximum	Minimum
WBC (count/mm^3^)	9197.5±3.6637	13500	2500
Hemoglobin (g/dl)	11.1±7.3	18.1	9.6
Platelets (*1000/mm^3^)	234.9±155.4	392	85
AST (IU/L)	88.8±27.8	49	13
ALT (IU/L)	57.10±22.10	66	9
ALP (IU/L)	544.3±66.46	1650	117
Bilirubin total (mg/dl)	1.61±0.1	3.6	0.3
Direct bilirubin (mg/dl)	1.7±0.1	0.9	0.1
Albumin (g/dl)	4±3.4	5.2	2.6
GGT (IU/L)	23.3±17.6	176	3
PT (Second)	12.1±1.6	17	11
INR	1±0.8	1.59	1
Triglycerides (mg/dl)	98.50±37.66	329	34
Cholesterol (mg/dl)	139.39±81.15	282	12
Glucose (mg/dl)	89.8±8.5	124	68

There was no significant relationship between patient FS score and allograft type (*p* = 0.79), underlying liver disease (*p* = 0.36) or familial relationship between donor and recipient (*p* = 0.64). The FS score was also not correlated with the type of immunosuppressive drug, i.e. tacrolimus (*p* = 0.51), sirolimus (*p* = 0.26), prednisolone (*p* = 0.49), and mycophenolate (*p* = 0.2). The CAP score was not significantly correlated with either of the above-mentioned parameters as well. 

**Figure 2:**
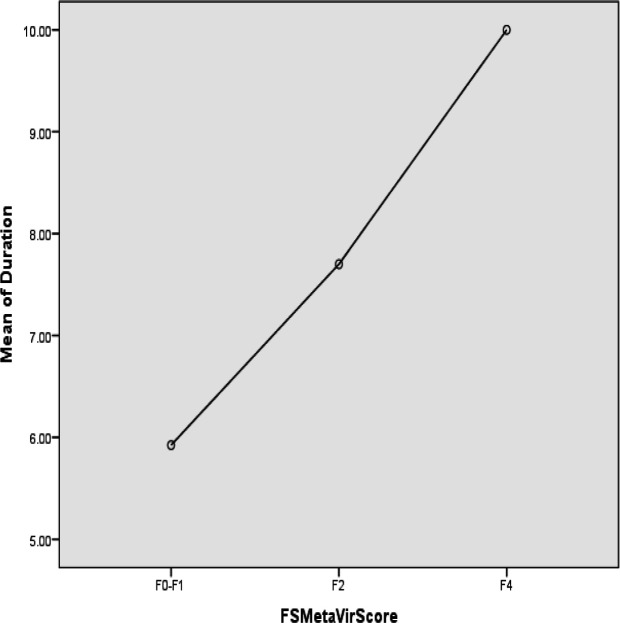
Correlation between Metavir score and the time after liver transplant

The fibrosis severity based on Metavir score was not associated with white blood cell count (*p* = 0.59), hemoglobin (*p* = 0.41), platelet count (*p* = 0.72), ALP (*p*=0.4), direct bilirubin (*p* = 0.42), GGT (*p* = 0.88), PT (*p* = 0.08), triglycerides (*p *= 0.08), or allograft type (*p *= 0.7). However, Metavir score was significantly and positively correlated with AST (*p* = 0.01), total bilirubin (*p* = 0.01), INR (*p* = 0.004), and cholesterol (*p* = 0.001). On the other hand, albumin (*p* = 0.004) and glucose (*p* = 0.003) levels were significantly and negatively correlated with Metavir score. The Metavir score and time of transplant were also positively and significantly correlated with each other (Figure 2, *p* = 0.03).

## Discussion

LT in children has a success rate of over 80% and a survival rate of twenty years. Most pediatric liver diseases are potentially treatable with LT. However, a number of patients experience a gradual decline in allograft function after LT (16). Histological abnormalities are often observed in allograft biopsies at 12 months after LT in pediatric recipients. In TE, the ultrasound wave velocity is used to measure LS. This technique is well tolerated and widely available; however, the equipment is expensive, a wide range of probes are needed, the measurement is inaccurate in patients who are obese or have mild fibrosis, and reproducible measurements are not possible in 20% of patients (17). Meanwhile, TE has been introduced as an accurate predictor of advanced fibrosis in children. Some pediatric studies have compared TE with post-transplant histology and have confirmed the validity of this method (10). Liver biopsy is currently considered the gold standard for the evaluation of liver fibrosis. However, it is an aggressive, painful, and potentially life-threatening procedure with limited repeatability in asymptomatic patients. Therefore, the development and validation of noninvasive tests that can accurately reflect the extent of liver fibrosis and cirrhosis are needed. Recent reports have shown that measurement of LS using Fibro-Scan allows accurate prediction of hepatic fibrosis (18). In their study, Barrault et al. indicated a high diagnostic accuracy for transient elastography in diagnosing liver fibrosis after LT with an area under the ROC curve (AUROC) of 0.83 (15). In a more recent study, Lee et al. demonstrated that the liver stiffness measurement was significant in pediatric patients with METAVIR ≥ F2 after LT, which is consistent with the results of the present study. They also suggested TE as a useful method for evaluating fibrosis after liver transplantation (19). Costa et al. also reported an AUROC of 0.86 for LE in predicting liver fibrosis (20). The current study demonstrated mild fibrosis in 85.2% and moderate fibrosis in 9.3% of patients and severe fibrosis in 5.6% of LT cases. The findings of Lee et al. regarding fibrosis grading are consistent with the current study, reporting 79% mild, 16% moderate, and 5% severe fibrosis (19). In addition, the results of the present study showed that fibrosis grade was significantly and positively associated with AST, total bilirubin, cholesterol, and INR while being negatively correlated with glucose and albumin levels. In addition, LS scores were significantly correlated with AST (*p* = 0.01), total bilirubin (*p* = 0.002), albumin (*p* = 0.001), PT (*p* = 0.03), INR (*p* = 0.001), glucose (*p* = 0.002), and the time between LT and TE (*p* = 0.02). The current results are consistent with the findings of Rigamonti et al. and Foucher et al., who both found a significant relationship between the degree of LS and AST, ALT, GGT, total bilirubin, platelet, PT, and albumin (18, 21). According to the current results, the most common underlying liver diseases among LT patients were Wilson disease and biliary atresia; however, no significant relationship was observed between underlying liver disease or degree of LS and severity of liver fibrosis, which is in line with previous studies. It was also found that only 14.9% of patients had moderate to severe fibrosis after one year, while Lee et al. reported a rate of 21%. Consistent with the present study, one research reported that the most common underlying diseases were biliary atresia, metabolic diseases, Wilson disease, and α1-antitripsin deficiency, and 20% of the patients had moderate to severe LS (22). Tomita et al. also showed that the most common underlying disease in patients was biliary atresia, and most patients also had mild LS and liver fibrosis (5). The current study showed no significant association between type of immunosuppressive drug (i.e. tacrolimus, sirolimus, prednisolone, mycophenolate) and Metavir score as well as LS. The results of Rigamonti et al. showed that the most common immunosuppressive therapy in LT patients was docetaxel/doxorubicin/cyclophosphamide (21), while tacrolimus was the most commonly used drug in the present study. In line with the results of the current study, Rigamonti et al. also showed that there was no significant relationship between type of immunosuppressive regimens and allograft histopathology after LT (21).

The present study demonstrated that 14.9% of LT patients had a METAVIR ≥ F2. The time between LT and TE was significantly correlated with LS and degree of liver fibrosis based on Metavir score. However, there was no significant relationship between LS and allograft type or underlying liver disease.

## Conflict of interests

The authors declare that they have no conflict of interest.
